# Gene Flow Across Large Distances in the Cavity‐Nesting Wasp *Deuteragenia subintermedia* in a Central European Forest

**DOI:** 10.1002/ece3.71294

**Published:** 2025-04-18

**Authors:** Laura‐Sophia Ruppert, Michael Staab, Nolan J. Rappa, Julian Frey, Gernot Segelbacher

**Affiliations:** ^1^ Chair of Wildlife Ecology and Management Albert‐Ludwigs‐Universität Freiburg Freiburg Germany; ^2^ Institute of Ecology Leuphana University of Lüneburg Lüneburg Germany; ^3^ Chair of Nature Conservation & Landscape Ecology Albert‐Ludwigs‐Universität Freiburg Freiburg Germany; ^4^ Department of Wildlife, Fish and Environmental Studies Umeå Sweden; ^5^ Chair of Forest Growth and Dendroecology Albert‐Ludwigs‐Universität Freiburg Freiburg Germany

## Abstract

Habitat connectivity and maintaining gene flow between populations is central for long‐term population persistence and is an essential element in conservation planning. However, data on dispersal ability and genetic population structure is lacking for almost all insect species. We here investigate if forest localities in the temperate, central European Black Forest are connected by gene flow. For this, we used partial genome sequencing on specimens of the solitary cavity‐nesting wasp *Deuteragenia subintermedia* (Hymenoptera, Pompilidae), a forest specialist that primarily nests in deadwood. We assumed that spatially uneven availability of standing deadwood has led to genetic substructuring. Contrary to our expectations, we did not find signs of population structure either on a regional or an individual level. Hence, for this solitary wasp species, dispersal seems not to be restricted across the Black Forest study sites (approximately 90 km distance) and none of the investigated environmental variables impacted genetic connectivity.

## Graphical abstract

Retention forestry is widely considered a promising approach to conserve forest biodiversity, and beneficial effects of more heterogeneous forest structure have been shown for several taxa. However, it is often unclear if the approaches taken locally can provide connectivity for specimens over the landscape or if they lead to “biodiversity islands” with little to no connection among each other. In this study, we tested if forest structural retention elements, especially standing deadwood, are distributed closely enough in the landscape of the Black Forest to allow connectivity of deadwood‐dependent species by investigating if a common spider‐hunting forest wasp, D. subintermedia, shows signs of isolation and structured populations across approximately 90 km of forest areas separated by open landscapes.

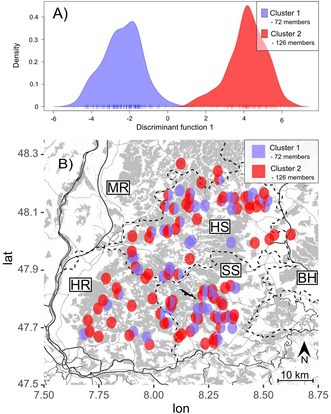

## 1 Introduction

A decline in the number of insects has been reported by several recent studies for different habitats and scales and is often linked to intensive land use (Forister et al. 2021; Seibold et al. 2019; Wagner 2019), for example by agriculture. However, the decline of insects is also found in forests (Staab et al. 2023), where human land use, for example, due to forestry interventions, is infrequent compared to agricultural land, which usually gets multiple interventions per year. Many different drivers are assumed to influence the decline of insects, of which one is the loss of connectivity between populations (Haddad et al. 2015; Keller et al. 2012; Manel and Holderegger 2013; Török et al. 2022) due to the fragmentation of the previously connected landscape into smaller habitat patches. This fragmentation has the potential to disrupt gene flow between populations within anthropogenically shaped landscapes and is therefore increasingly being considered in conservation planning (Hansen et al. 2018; Lecocq et al. 2018).

To understand the influence of the quality of the remaining habitat patches and the surrounding landscape on the diversity and abundance of insects, several studies have been conducted in agricultural areas with a focus on flower visiting insects (e.g., Kleijn and van Langevelde 2006; Cano et al. 2022; Hall et al. 2022). For forest‐dwelling insects, many studies focused on woody patches surrounded by open landscapes, finding both negative and neutral responses of, for example, bee and wasp diversity to increasing distance between habitat patches (Gobatto et al. 2023; Schüepp et al. 2011).

However, some effects of large distances between habitat patches on populations may not directly result in measurable decline of abundance or diversity in the short term, while still holding the potential to affect long‐term population health. One such effect is genetic erosion, which is known to occur on isolated islands due to insufficient gene flow (Forsdick et al. 2017; Vandewoestijne et al. 2008; Yan et al. 2023). The most common effects of genetic erosion in small populations tend to be increased rates of inbreeding and proneness to experience genetic drift, which can both cause erosion of genetic diversity (Bosse and van Loon 2022; Forsdick et al. 2017; Reed and Frankham 2003), and thus potentially reduced fitness due to inbreeding depression and accumulation of deleterious mutations in the long term (Bosse and van Loon 2022; Reed and Frankham 2003; Vandewoestijne et al. 2008; Vrijenhoek 1994). Importantly, these effects of genetic erosion are expected to occur before effects on species abundance can be observed (Pflüger et al. 2019). This underlines the need to investigate and ensure genetic connectivity of populations within habitat patches for long‐term population health (Auffret et al. 2015; Hansen et al. 2018; Lienert 2004; Reed and Frankham 2003; Whitla et al. 2024).

Genetic connectivity and hence gene flow are maintained by the dispersal of individuals of a species (Franklin 1980), which is a highly individual trait between and within species. For solitary bee species inhabiting sandy grasslands, foraging distances varying between 100 m and 1400 m have been observed (Zurbuchen et al. 2010), while some tropical orchid bees can have dispersal distances up to 23 km or more (Janzen 1971; Wikelski et al. 2010). For the majority of solitary bee and wasp species, dispersal and foraging distances are, however, unknown. Hence, investigating genetic connectivity or isolation of populations has become a viable workaround to investigate population connectivity in fragmented landscapes (Manel and Holderegger 2013; Pfeiler and Markow 2017), which can provide insights into the dispersal abilities of species and if the landscape is configured in a way that allows dispersal between sites. For this, partial genome sequencing—such as restriction site associated DNA sequencing (RAD; Miller et al. 2007)—has become established as an affordable and effective method to investigate species gene flow of the past and present and the resulting population structures (e.g., Klinga et al. 2019; Storfer et al. 2018).

A species with little information on its dispersal distances available is *Deuteragenia subintermedia* (Hymenoptera: Pompilidae), a common spider‐hunting forest specialist that nests primarily in dead wood (Day 1988; Schmid‐Egger and Wolf 1992). It has been previously observed that the forest specialist bee and wasp community in the Black Forest, of which *D. subintermedia* is a part showed increased abundance in forests with a high amount of standing deadwood and high stand structural complexity (Rappa et al. 2023). Attempts to make deadwood more widely available in the Black Forest are ongoing; however, the dedicated efforts have only started recently (Alt‐ und Totholzkonzept, ForstBW 2016). Additionally, due to the black forest being a mosaic of differently managed areas (Storch et al. 2020), the availability of suitable habitat with large volumes of resources like deadwood varies strongly in the landscape matrix, creating the need to investigate if the distribution is continuous enough to allow uninhibited dispersal of deadwood‐dependent species.

To test if forest structural retention elements, especially standing deadwood, are distributed closely enough in the landscape of the Black Forest to allow connectivity of deadwood‐dependent species, we here investigate if a common spider‐hunting forest wasp *D. subintermedia* shows signs of isolation and structured populations across approximately 90 km of forest areas separated by open landscapes. We hypothesise that genetic structure in *D. subintermedia* will vary in response to the availability of dead wood and thus nesting sites across the landscape.

## 2 Materials and Methods

### 2.1 Research Area

This study was part of the ‘Conservation of forest biodiversity in multiple‐use landscapes of Central Europe’ (ConFoBi) framework (Storch et al. 2020). The ConFoBi project investigates how structural retention forestry approaches in the southern Black Forest (Baden‐Württemberg, Germany) influences several aspects of biodiversity. A location map of the whole ConFoBi research area can be found in Supporting Information 6. The study area is a continuous cover forest ranging over 5000 km^2^ with 75% of the landscape covered by forest, which is primarily coniferous with planted Norway spruce ( *Picea abies* L.), which would naturally not occur in high density, and native silver fir ( *Abies alba* Mill.). Deciduous forest consists mostly of European beech ( *Fagus sylvatica* L.), with sycamore maple ( *Acer pseudoplatanus* L.) and sessile oak ( *Quercus petraea* L.) making up a smaller part. Since 2010, forestry is practiced under the guidelines of a state‐wide retention program. Within this area, 135 study plots with 1 ha area were selected in state‐owned forest in 2016. The plots were selected to represent two gradients: (1) the number of snags detected in aerial images, ranging from zero to more than 20 snags per ha; (2) the forest cover of the surrounding landscape (circular, 2500 ha), comprising a gradient from low (3.0%) to high (92.2%) landscape‐level forest cover. The minimum distance between neighbouring plot centres is 750 m. The plots can be subdivided into the geographic areas Hochschwarzwald, Südschwarzwald, Hochrhein, Mittleres Rheintal und Baar/Hegau, which is a mostly historical grouping. The research area is further divided into forest districts, for which the same state level guidelines apply, that is, the “Old and Dead Wood programme” (Alt‐ und Totholzkonzept, ForstBW 2016) but are otherwise mostly managed independently from each other. The Forest districts can therefore show prominent differences in, for example, the amount of standing or lying deadwood and are not guaranteed to have continuous forest cover connecting them. In the final dataset 27 forest districts were represented. (Storch et al. 2020).

### 2.2 Focal Species

*Deuteragenia subintermedia* is part of the family Pompilidae, whose larvae are spider‐hunting or ectoparasites of spiders (or, rarely, kleptoparasites on other Pompilidae) whereas the adults feed on pollen and nectar. Pompilidae are thus ecologically important as predators and pollinators. *Deuteragenia subintermedia* is a common spider‐hunting forest wasp, known to prey, for example, on spiders of the families Clubionidae, Salticidae and Segestriidae. Female individuals will drag the spider to a suitable cavity—such as abandoned galleries of wood‐boring beetles in standing deadwood—where they will deposit the spider in a brood‐cell with one egg attached and seal the brood‐cell with spiderweb and plant material. Several brood‐cells can be built adjacent to each other in one cavity (Schmid‐Egger and Wolf 1992). A picture of *Deuteragenia subintermedia* nesting cells is available in Supporting Information 6. Studies investigating the effects of deadwood largely focused on deadwood‐dwelling species such as saproxylic beetles (e.g., Lassauce et al. 2011; Plath and Fischer 2024). However, several species that do not dwell in deadwood for their whole lifecycle are still largely dependent on the availability of deadwood and thus need to be investigated for informed conservation planning. We chose *D. subintermedia* as our focal species, as it is dependent on deadwood for nesting but is also expected to be able to forage and potentially disperse beyond deadwood patches and is therefore suitable to investigate specialised and mixed resource dependencies. Only male specimens were chosen for sequencing because, in Hymenoptera, males have a haploid genome, unlike females, which are diploid. Thus, using the same sequencing depth overall, the haploid genomes per individual could be sequenced with higher depth. The average foraging distance of *D. subintermedia* is not known, but based on the measured foraging distances of other cavity‐nesting Hymenoptera (Gathmann and Tscharntke 2002; Pufal et al. 2017; Zurbuchen et al. 2010) it can be assumed to be between a few hundred metres to a maximum of a few kilometres.

Specimens of *D. subintermedia* were collected using trap nests (Staab et al. 2018) on 134 study plots (one plot of the original 135 was impassable during sampling). The traps were installed in March 2020 and collected in October 2020. On each of the study plots, trap‐nests were secured in pairs to wooden poles ~1.5 m high, at approximately halfway between northwest and southeast plot corners, totalling four traps per plot (see Rappa et al. 2023). After trap retrieval in autumn, all collected nests were placed in a cooling chamber at 4°C until February. After this simulated winter diapause, the specimens hatched and were preserved. The first live *Deuteragenia* sp. specimen of every *Deuteragenia* nest in every trap tube was collected directly into 100% ethanol and stored at −20°C. Prior to DNA extraction, the specimens were removed from the freezer only once to morphologically identify specimens and ensure that only males were included. We aimed to use two *D. subintermedia* specimens per plot, one from the northwest corner and one from the southeast corner. If specimens were not available on both corners, we picked two from the same trap from different nests. This bears the risk that these specimens might be the offspring of the same mother who built several nests in one trap nest. In total, we used 199 *D. subintermedia* specimens. With this set of samples, 33 plots were only represented by one sample, 22 plots had 2 specimens from the same trap location and 61 plots had two specimens from two locations. Hence, 116 plots of the total plot selection were represented, and 19 plots of the total plot selection were not represented in our data, because the target species did not occur.

### 2.3 DNA Extraction and Sequencing

DNA of all specimens was extracted with Qiagen DNeasy Blood & Tissue Kit (Qiagen, Hilden, Germany) with a standardised protocol following the manufacturers' recommendations (Supporting Information 1). After DNA extraction, concentration was measured on a Qubit 4 Fluorometer with the corresponding Qubit dsDNA HS Assay Kit (Thermo Fisher Scientific, Waltham, MA, USA). Samples were stored at −20°C until further processing. Other *Deuteragenia* species present in the study area are *Deuteragenia bifasciata* and *Deuteragenia variegata*. Thus, to ensure that only specimens of *D. subintermedia* were analysed, we ran a PCR on the COI barcode (PCR protocol in Supporting Information 2) using the degenerated Meyer Primers dgLCO1490 (5′ GGT CAA CAA ATC ATA AAG AYA TYG G 3′) and dgHCO2198 (5′TAA ACT TCA GGG TGA CCA AAR AAY CA 3′; Meyer 2003). The PCR product was sent for Sanger sequencing at Microsynth (Balgach, Switzerland) to confirm morphological species identifications. As one sample was genetically identified as *D. variegata* , it was excluded from all further analyses. The remaining DNA samples were sent for RAD sequencing at Floragenex (Beaverton, Oregon, USA). The RAD sequencing involved preparation of RAD libraries by single digestion, multiplex RAD adaptor ligation, size selection, and PCR amplification and product purification. Sequencing was done with 1 × 100 bp on Illumina HiSeq. The raw sequencing data were processed in the ipyrad pipeline (Eaton and Overcast 2020). Data were demultiplexed and reads filtered based on quality scores. This involved base trimming from the 3′ end if the quality score was consistently below 20 and removal of reads with more than 5 Ns. Additionally, the reads were searched for adapters and their reverse complement and trimmed (with a cutadapt code implementation) if adapters were found. If trimmed reads went below a minimum length of 35 bp (default ipyrad value) they were discarded. Afterwards, reads were dereplicated and denovo clustered within samples. The sequence similarity threshold to consider reads to have come from the same locus was set to 0.85. Due to the presence of Ns, indels, sequencing errors or polymorphisms, a value of 0.85–0.9 is recommended to ensure homologous sequences clustering together (Eaton and Overcast 2020). During the next step, sequencing error rate and heterozygosity were estimated and used to call the consensus of sequences to distinguish “real” haplotypes from sequencing errors. The minimum depth at which statistical base calls were made was left at the default value of 6. To account for uneven sequencing coverage and overrepresentation of repetitive regions of the genome, the maximum depth above which clusters were excluded was set to 10,000. Because we worked with haploid male specimens, the maximum allowed allele consensus was set to 1. The maximum fraction of uncalled bases (N) in the consensus sequences was left at the default value of 0.05 to avoid clustering problems between samples in the following step. Clustering between samples was again done based on sequence similarity with a threshold of 0.85. Finally, the clustered data were filtered before being written to output files. The maximum number of Single nucleotide polymorphisms (SNPs) allowed in a final locus was set at 0.2 to remove poor alignments in repetitive regions with many N‐SNPs. Also, the maximum number of indels allowed in a final locus was set at 5 to remove poor alignments, and the maximum number of polymorphic sites in a locus was set at 0.25 to detect potential paralogs.

### 2.4 Environmental Distance

We calculated the environmental distance as a Euclidean distance matrix of the plots with a selection of environmental variables characterising the forest habitat and additionally using only the value of canopy closure for a second Euclidean distance matrix. The concept of environmental distance is an extension of the concept of isolation by distance. While isolation by distance expects the genetic distance between populations to be proportional only to the geographic distance, environmental distance expects the genetic distance between populations to be proportional to the similarity of their environment (Bradburd et al. 2013; Brown and Kodric‐Brown 1977; Sexton et al. 2014).

The environmental distance was calculated based on the average elevation of the study plots (m a.s.l., derived from a digital terrain model provided by the State Agency of Spatial Information and Rural Development of Baden‐Württemberg, lgl‐bw.de, 2005), the mean diameter of trees at breast height > 7 cm (DBH) and the sum of the basal area of trees calculated from data of a full forest inventory in 2017. We also included the volume of lying and standing deadwood with deadwood sampling conducted along V‐transects on each plot, measuring all deadwood structures > 7 cm DBH and a minimum height of 1.3 m at the upper slope. Calculated from the same forest inventory data, we also included the percentage of coniferous trees on a plot. Furthermore, from remote sensing data, we included the canopy closure calculated as the percentage of plot surface lower than 3 m above ground and the effective number of layers derived from Terrestrial Laser Scanner LiDAR data, which calculates the number of 1‐m thick layers of foliage weighted by how much they are filled (Ehbrecht et al. 2016). Additionally, we included the forest cover in the surrounding 10 km^2^ (1000 ha/10 km^2^ moving window on Landsat land cover data by Landesanstalt für Umwelt Baden‐Württemberg LUBW 2010). The environmental variables were centered and scaled (mean = 0, SD = 1), tested for Kendall correlation, and if necessary, log‐transformed (lying deadwood volume) or square‐root transformed (standing deadwood volume) to increase normality and homogeneity prior to the calculation of the Euclidean distance matrix.

### 2.5 Data Analysis

The relationship between the abundance of *D. subintermedia* per plot and the same environmental variables as for the environmental distance was analysed using a generalised linear model (GLM) with a negative binomial distribution. Additionally, a negative binomial generalised linear mixed model (GLMM) with regions as a random factor was calculated, and an averaged model based on negative binomial GLM was obtained from model selection using the Akaike Information Criterion, retaining models with a delta AIC < 2. The result of the GLMM is available in Supporting Information 4, and of the averaged model in Supporting Information 5.

We performed a hierarchical analysis of molecular variance (AMOVA, 1000 permutations, allowed level of missing data = 0.05) and calculated the pairwise FST in Arlequin 3.5.2 (Excoffier and Lischer 2010) to investigate genetic variation between the geographic areas Hochschwarzwald, Südschwarzwald, Hochrhein, Mittleres Rheintal and Baar/Hegau and also the 27 forestry districts. Out of the total 551,976 bases in the original sequence, 253,082 bases passed the allowed level of missing data. Of these, 129,713 bases contained data for all defined populations and were used in the distance computation by region. For the forestry districts, 115,616 bases contained data for all defined populations.

We conducted individual‐based analysis without a priori inferred populations to test for small scale genetic variation. We calculated Mantel tests (based on Spearman correlation, 9999 permutations) for the genetic distance (Bray‐Curtis distance), the geographic distance (Euclidean distance) and environmental distance (Euclidean distance) using the R packages geodist (Padgham et al. 2021), ape (Paradis and Schliep 2019) and vegan (Oksanen et al. 2020). We calculated pairwise Moran's I statistic (Hardy and Vekemans 1999) (1) in intervals of 10 km, (2) for 10 distance classes with automatic interval selection for even distribution of pairs and (3) in intervals of 1 km up to 10 km, using SPAGeDI regression analysis (Hardy and Vekemans 2002, 200 Permutations). A linear two‐way ANOVA was used to test the influence of geographic distance on Moran's I.

To find the most likely number of genetic clusters, we calculated a clustering approach using Discriminant Analysis of Principal Components in adegenet (DAPC, Jombart et al. 2010) by running successive K‐means, without assuming a panmictic population. Finally, we conducted an individual‐based STRUCTURE analysis to investigate population structure based on allele frequencies, with 100,000 burn‐ins and 1,000,000 repetitions, assuming panmixis and unlinked markers. From data processing, 20,687 SNPs were recovered. Of these 9944 were present in 90% of the samples and 2953 were unlinked. The STRUCTURE population estimations were investigated using the mean log‐normal probability with the STRUCTURE Harvester program (Earl and vonHoldt 2012) and Evanno statistics (Evanno et al. 2005).

## 3 Results

### 3.1 Environment and Geographic Distance

The abundance of *D. subintermedia* declined with increasing canopy closure (GLM, z = −2.160, *p* = 0.031, Table 1), whereas none of the other tested environmental variables influenced abundance. The same result was found in the alternative analyses applying a generalised linear mixed model (Supporting Information 4) or model averaging (Supporting Information 5) Genetic distance was, however, not related to environmental distance characterised by the same set of environmental variables (Mantel test, *r* = 0.022, *p* value = 0. 227) or by canopy closure only (Mantel test, *r* = −0.010, *p* value = 0.646). Additionally, there was no relationship between the genetic distance among individuals and the geographic distance (Mantel test, *r* = 0.026, *p* value = 0.117).

**TABLE 1:** Summary statistics of the negative binomial GLM for the abundance of *Deuteragenia subintermedia* and the selected environmental variables.

Environmental variable	Estimate	Std. error	*z*‐value	*p* value
Average DBH	0.005	0.007	0.750	0.456
Average elevation	−0.009	0.007	−1.330	0.185
Basal area	0.003	0.070	0.040	0.098
**Canopy closure**	**−0.135**	**0.0625**	**−2.160**	**0.031**
Effective number of layers	0.000	0.064	0.000	0.999
Forest cover 10 km^2^	−0.050	0.057	−0.870	0.385
Lying deadwood (log)	0.036	0.089	0.620	0.533
Percentage coniferous	0.100	0.065	1.550	0.120
Standing deadwood (sqrt)	0.055	0.054	1.000	0.315

**FIGURE 1:**
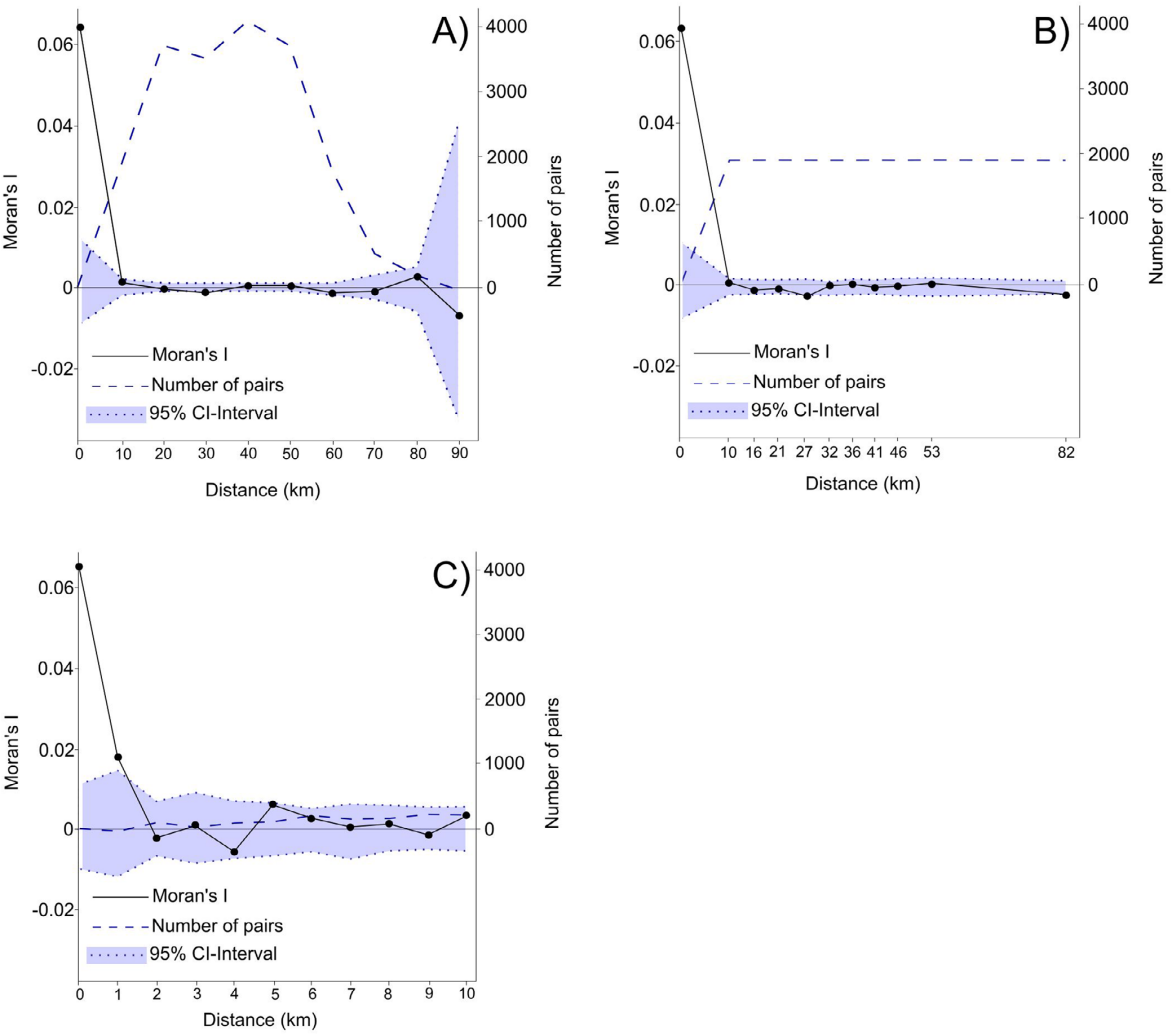
Moran's I (Hardy and Vekemans 1999), plotted with the uninterrupted line and values shown on the left *y*‐axis, and the corresponding number of pairs, plotted with the blue dashed line and values shown on the right *y*‐axis, with upper and lower limit of 95%‐confidence (CI) interval shown with the dotted lines and blue background. (A) Calculation for distance classes in intervals of 10 km each. (B) Calculation for 10 distance classes automatically selected to contain even numbers of pairs. (C) Calculation for 10 distance classes in intervals of 1 km up to 10 km. No influence of distance on the kinship coefficient was found.

The independence of the genetic variation between individuals towards distance was confirmed by the pairwise relationship estimation using Moran's I. The number of pairs for the calculation of the Moran's I ranged between 0 (90–100 km distance) and 4060 (30–40 km distance). The Moran's I statistic for predetermined intervals of 10 km (see Figure 1A) ranged from 0.064 (no distance, outside of 95% confidence (CI) interval) to −0.0069 (80–90 km distance) with no significant deviations from 0, whereas 0 represents a random distribution. Similar results were obtained for the distance intervals automatically selected to contain even numbers of pairs (see. Figure 1B). Finer scale analysis in intervals of 1 km up to 10 km did not show significant deviations from a random distribution of genetic variation (see Figure 1C).

Similar results as for the individual based analyses were observed for the AMOVA using groupings by region and forest district. Both, the comparison of molecular diversity grouped into to the regions Hochschwarzwald, Südschwarzwald, Hochrhein, Mittleres Rheintal and Baar/Hegau and the grouping into forestry districts showed only minimal molecular variation among the regions and districts. For the regional grouping, 99.63% of the observed genetic variation was within regions, and variation among regions only contributed a very small amount of total genetic variation (FST = 0.004, *p* = 0.006) (Table 2). The grouping into forest districts found 98.841% of the genetic variation to be within districts, and among districts with an FST of 0.01159 (*p* ≤ 0.001; Table 2).

**TABLE 2:** Summary of AMOVA results for the comparison by Black Forest region (Hochschwarzwald, Südschwarzwald, Hochrhein, Mittleres Rheintal und Baar/Hegau) and forest districts.

Source of variation	Sum of squares	Variance components	Percentage variation	*p* value
Black Forest region				
Among regions	1319.538	1.174	0.370	0.006
Within regions	51312.827	315.850	99.630	
Total	52632.365	317.024		
Forest districts				
Among districts	7200.352	3.006	1.159	< 0.0001
Within districts	41252.203	256.449	98.841	
Total	48452.555	259.456		

### 3.2 Population Structuring

**FIGURE 2:**
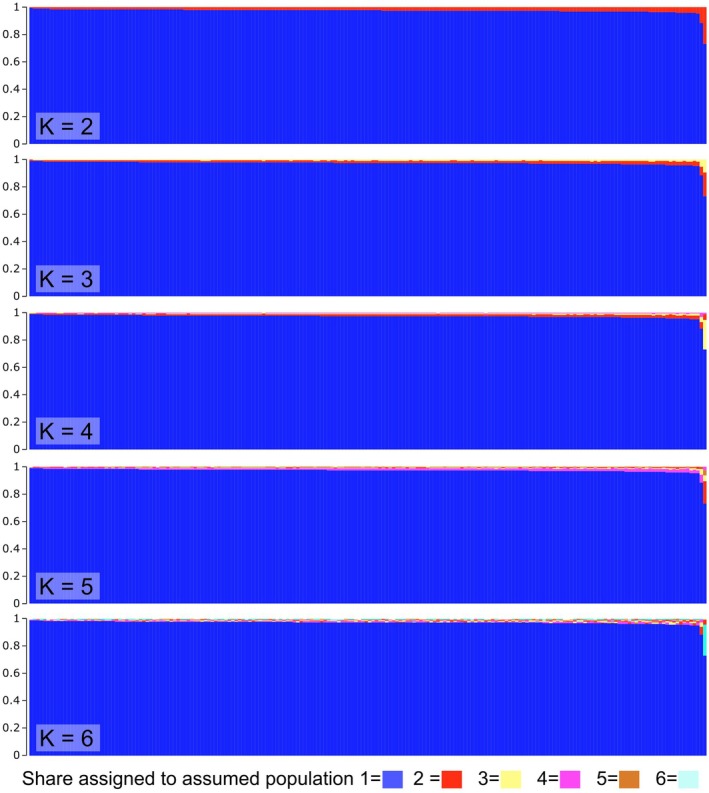
Visualisation of STRUCTURE results for *K* = 2 to *K* = 6, whereas *K* is the number of assumed populations. Specimens are represented as one bar each, for better overview the specimen labels are not shown. The majority of genetic variation is explained by one population, which is assigned the blue colour at the lower fraction of the bars, with only minimal shares being assigned to the assumed additional populations, which can be seen in the red or multicoloured fractions at the top of the bars.

Genetic variation was not only independent from environment and distance but also did not show any substructure of the population. Visual inspection of the STRUCTURE results suggests that only a small genetic fraction varies between individuals (Figure 2), indicating that the true K equals a single population. The highest delta *K* was found for *K* = 2 populations (Table 3); however, the delta *K* calculated after Evanno et al. (2005) is numerically not able to find *K* = 1 as the best value.

**TABLE 3:** Results of Evanno statistics (Evanno et al. 2005) for STRUCTURE results on 198 *Deuteragenia subintermedia* individuals.

*K*	Reps	Mean LnP (*K*)	Stdev LnP (*K*)	Ln’ (*K*)	|Ln” (*K*)|	Delta *K*
1	3	−244024.700	14.212	—	—	—
**2**	**3**	**−241625.967**	**67.708**	**2398.733**	**2454.767**	**36.255**
3	3	−241682.000	27.719	−56.033	163.167	5.886
4	3	−241574.867	41.597	107.133	149.567	3.596
5	3	−241617.300	45.310	−42.433	179.333	3.958
6	3	−241480.400	332.909	136.900	—	—

*Note:* Marked in bold is the value for *K* with the highest Delta *K*.

**FIGURE 3:**
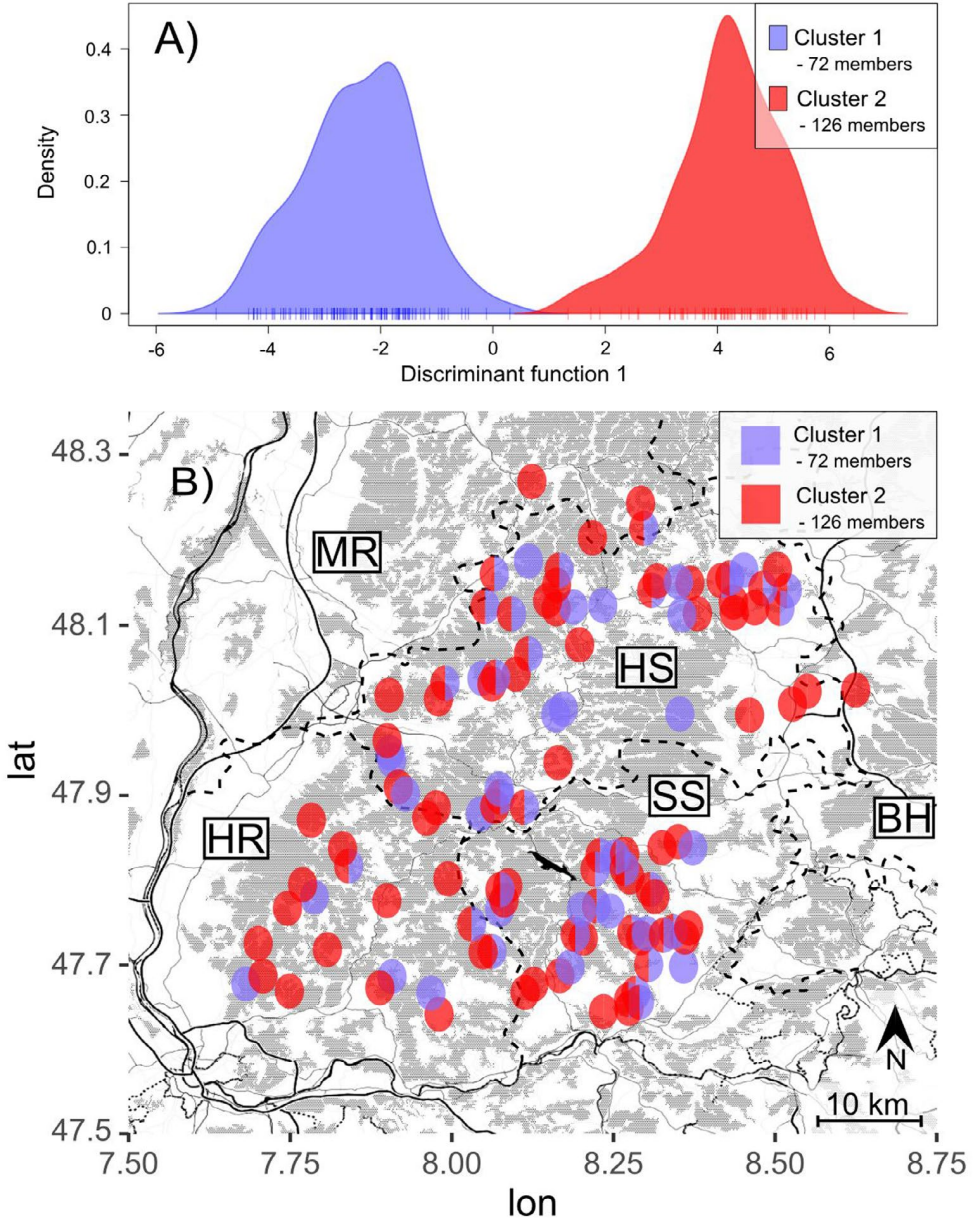
(A) DAPC Clustering found *K* = 2 populations to explain the observed genetic variation best. Of the sequenced specimens 72 were assigned to Cluster 1 and 126 to Cluster 2. (B) The plotted results of the unsupervised clustering did not show a geographic pattern. Forest areas are shown in grey. The dashed lines indicate the borders of the forest districts Südschwarzwald (SS), Hochschwarzwald (HS), Mittleres Rheintal (MR), Hochrhein (HR) and Baar/Hegau (BH).

In contrast, the unsupervised clustering approach found that based on the Bayesian Information Criterion (BIC) the data is best explained by *K* = 2 (Supporting Information 3), though the “bend” of the graph was only weakly visible. We thus argue that we have one unstructured population representing *K* = 1. As seen in Figure 3 for *K* = 2, the specimens were distributed with 72 specimens in one cluster and 126 in another cluster. However, the plotted population assignments did not show a clear geographic pattern.

## 4 Discussion

*Deuteragenia subintermedia* in the Black Forest showed no signs of isolation or pronounced spatial genetic structures, suggesting that gene flow is maintained across our research area. In our study, the abundance of *D. subintermedia* declined with increasing canopy cover but was not influenced by other tested variables, such as standing and lying deadwood volume, which are key targets of the retention forestry management in the black forest (Alt‐ und Totholzkonzept, ForstBW 2016). This may indicate that even though *D. subintermedia* is dependent on standing deadwood, in the Black Forest, the availability of light forest microhabitats with lower canopy cover, potentially created by natural disturbances (e.g., windthrow) is more limiting in terms of abundance than the availability of standing deadwood. This shows that even for resource‐specialised species, diversified habitat corridors need to be kept between habitat patches to enable gene flow. In this regard, it has been observed for species of the bee genus *Euglossa*, which have long dispersal ranges (Janzen 1971; Wikelski et al. 2010), that genetic structure was higher for species with higher resource specialisation and that the amount of intact habitat in between research sites positively influenced the gene flow among the sites (Hernandez and Suni 2024). In a further study on *Euglossa*, it was however shown that differentiation between research areas with distances of up to 80 km was low, suggesting high levels of gene flow (Suni et al. 2014) and emphasising the individuality of dispersal capability even within a genus. Therefore, even though the available resources between habitat patches seem to be sufficient to allow gene flow for *D. subintermedia*, closely related species such as *D. bifasciata* and *D. variegata* might show different results, due to slight variations in resource specialisation and individual dispersal capabilities.

Areas with large amounts of standing deadwood and high structural complexity, which could be considered habitat islands or patches for deadwood dependent organisms, although slowly increasing (Schall et al. 2021), are still rare within forests in Central Europe including our research area (compare e.g., https://bwi.info; 69Z1JI_L202of_2012_L203). Therefore, to allow gene flow, species will have to disperse between those areas, which not all species are equally capable of (Reim et al. 2018; Steyn et al. 2016). As a flying species, *D. subintermedia* can be expected to be able to find and utilise resources in a larger radius than flightless species, as is also suggested by the study of Dussex et al. (2016), which found no genetic structuring for the flight capable form of an alpine stonefly as opposed to its flightless form, which did show genetic clustering between habitat streams. Similar results were found for a flightless carabid beetle by Gaublomme et al. (2012), showing that the fragmentation of a formerly connected forest landscape hampers gene flow and drives the species genetic differentiation. Nevertheless, flight capability alone is not always a good predictor of a species ability to emigrate between habitats, as between suitable habitat patches barriers to gene flow—such as urban areas—can hamper the dispersal of individuals, as it has been shown for the rare solitary bee species *Colletes floralis* in Ireland and Scotland (Davis et al. 2010). Our results do however indicate that even though based on its body size *D. subintermedia* would not be expected to be able to cross large dispersal distances, no strict barriers to gene flow seem to be present in the Black Forest, allowing it to disperse through the open landscape between the deadwood substrates it needs for nesting.

The Black Forest has a long history of management. However, approaches to increase habitat for deadwood specialists have only been implemented recently (Alt‐ und Totholzkonzept, ForstBW 2016) and for many species it is not yet clear if the current management has the intended benefits. Local abundance holds valuable information for the estimation of a species genetic potential because larger populations are expected to have a larger genetic diversity (Ellegren and Galtier 2016). However, insect decline has also been confirmed for forest insects (Staab et al. 2023). Thus, to ensure long‐term survival of species—such as cavity‐nesting Hymenoptera*—*in times of drastic environmental changes, stable connectivity of populations and consequently stable gene flow has to be considered in environmental planning. Investigation of the genetic connectivity of *D. subintermedia* has shown that some species can be resistant to land use in terms of maintaining healthy genetic population connectivity. We also found indications that even though *D. subintermedia* is dependent on standing deadwood for nesting, abundance was driven by canopy cover in our research area. This points out the need to consider specialist species with mixed resource dependencies or suites of environmental characteristics in the planning of conservation efforts.

*Deuteragenia subintermedia* shows little to no genetic population structure or signs of isolation effects in the investigated area of the Black Forest. However, as *D. subintermedia* is a commonly occurring forest specialist species, its genetic connectivity cannot directly inform on how well rare species with low population numbers and species with highly specific habitat requirements are connected in the Black Forest landscape. Therefore, further investigation is necessary to assess how well the currently implemented forest structural retention approach conserves biodiversity in the Black Forest. Thus, the genetic connectivity of additional species, representing a wider range of dispersal capabilities, rareness, and habitat requirements should be investigated.

## Author Contributions

**Laura‐Sophia Ruppert:** conceptualization (lead), formal analysis (lead), investigation (lead), methodology (lead), writing – original draft (lead), writing – review and editing (lead). **Michael Staab:** conceptualization (supporting), formal analysis (supporting), investigation (supporting), supervision (lead), writing – original draft (supporting), writing – review and editing (supporting). **Nolan J. Rappa:** conceptualization (supporting), methodology (supporting), resources (supporting), writing – original draft (supporting). **Julian Frey:** resources (supporting), writing – original draft (supporting). **Gernot Segelbacher:** conceptualization (supporting), supervision (equal), writing – original draft (supporting), writing – review and editing (supporting).

## Conflicts of Interest

The authors declare no conflicts of interest.

## Supporting information


Appendix S1.

## Data Availability

The data that support the findings of this study are openly available in Dryad at https://doi.org/10.5061/dryad.ns1rn8q4f.
