# Supplementary material for: Gene Flow Across Large Distances in the Cavity‐Nesting Wasp Deuteragenia subintermedia in a Central European Forest
Source: Ecol Evol. 2025 Apr 18;15(4):e71294. doi: 10.1002/ece3.71294 (PMC12007984; doi:10.1002/ece3.71294)
Supplement: Supplementary file 1 — Appendix S1. [file ECE3-15-e71294-s001.docx]

**Title**

Gene flow across large distances in the cavity-nesting wasp *Deuteragenia subintermedia* in a central European forest

**Authors**

Laura-Sophia Ruppert^1^, Michael Staab^2^, Nolan J. Rappa^3,4^, Julian Frey^5^, Gernot Segelbacher^1^

**Addresses**

^1^ Chair of Wildlife Ecology and Management, Albert-Ludwigs-Universität Freiburg, Stefan Meier Strasse 76, 79104 Freiburg, Germany

^2^ Institute of Ecology, Leuphana University of Lüneburg, Universitätsallee 1, 21335 Lüneburg, Germany

^3^ Chair of Nature Conservation & Landscape Ecology, Albert-Ludwigs-Universität Freiburg, Stefan Meier Strasse 76, 79104 Freiburg, Germany

^4^ Department of Wildlife, Fish and Environmental Studies, Skogsmarksgränd, 90736 Umeå, Sweden

^5^ Chair of Forest Growth and Dendroecology, Albert-Ludwigs-Universität Freiburg, Tennenbacher Str. 4, 79106 Freiburg, Germany

**Contents**

[**Supplementary data 1**: DNA extraction protocol for QIAGEN DNeasy Blood & Tissue Kit 3](#_Toc193371409)

[**Supplementary data 2**: PCR protocol for COI amplification with insect DNA 4](#_Toc193371410)

[**Supplementary data 3**: Bayesian Information Criterion 5](#_Toc193371411)

[**Supplementary data 4**: Summary statistics of negative binomial GLMM 6](#_Toc193371412)

[**Supplementary data 5:** Summary statistics of averaged model based on negative binomial GLM 7](#_Toc193371413)

[**Supplementary data 6:** Sampling overview 8](#_Toc193371414)

# **Supplementary data 1**: DNA extraction protocol for QIAGEN DNeasy Blood & Tissue Kit

Kit: QIAGEN DNeasy Blood & Tissue Kit (250) (Product No. #69506)

BEFORE STARTING: Preheat the incubator to 55-56°C. Before using some of the buffers provided in the kit for the first time, add the appropriate amount of ethanol (96%-100%) as indicated on the bottle to get a working solution. Per sample prepare 1 column, 2 collection-tubes and 1 final tube. Prepare glass with distilled water for used pestles. Prepare 100% Ethanol.

1. Place about 50mg of sample in a 1.5ml tube. Add 180µl Buffer ATL. Grind sample using a sterile plastic pestle each.
2. Add 20 µl Proteinase K, vortex briefly and spin down. Incubate at 56°C on the shaker for 1-3h.
3. For each sample, mix 200 µl Buffer AL with 200 µl 100% Ethanol.
4. Vortex samples for 15 sec and spin down. Add 400 µl AL/Ethanol mixture to each sample and vortex briefly
5. Centrifuge for 2 min @ 10´000 x g. Transfer the clear lysate into a DNeasy Mini spin column.
6. Centrifuge for 1 min @ 6´000 x g. Discard flow-through and transfer the column to a new collection tube.
7. Add 500 µl Buffer AW1 and centrifuge for 1 min @ 6´000 x g. Discard flow-through and transfer the column to a new collection tube.
8. Add 500 µl Buffer AW2 and centrifuge for 3,5 min. @ max. x g. Discard flow-through
9. Place the column in a new 1.5-ml micro-centrifuge tube and pipette 50-200 µl Buffer AE directly onto the membrane.
10. Incubate the columns at RT for 2min.
11. Centrifuge @ 10´000 x g for 1 min.
12. Store the DNA extractions at -20 °C.

# **Supplementary data 2**: PCR protocol for COI amplification with insect DNA

|  | Temperature °C | Time | Cycle number |
| --- | --- | --- | --- |
| Initial Denaturation | 95 | 3min |  |
| Denaturation | 95 | 30sec | 40 |
| Annealing | 55 | 30sec |  |
| Extension | 72 | 50sec |  |
| Final extension | 72 | 5min |  |

**
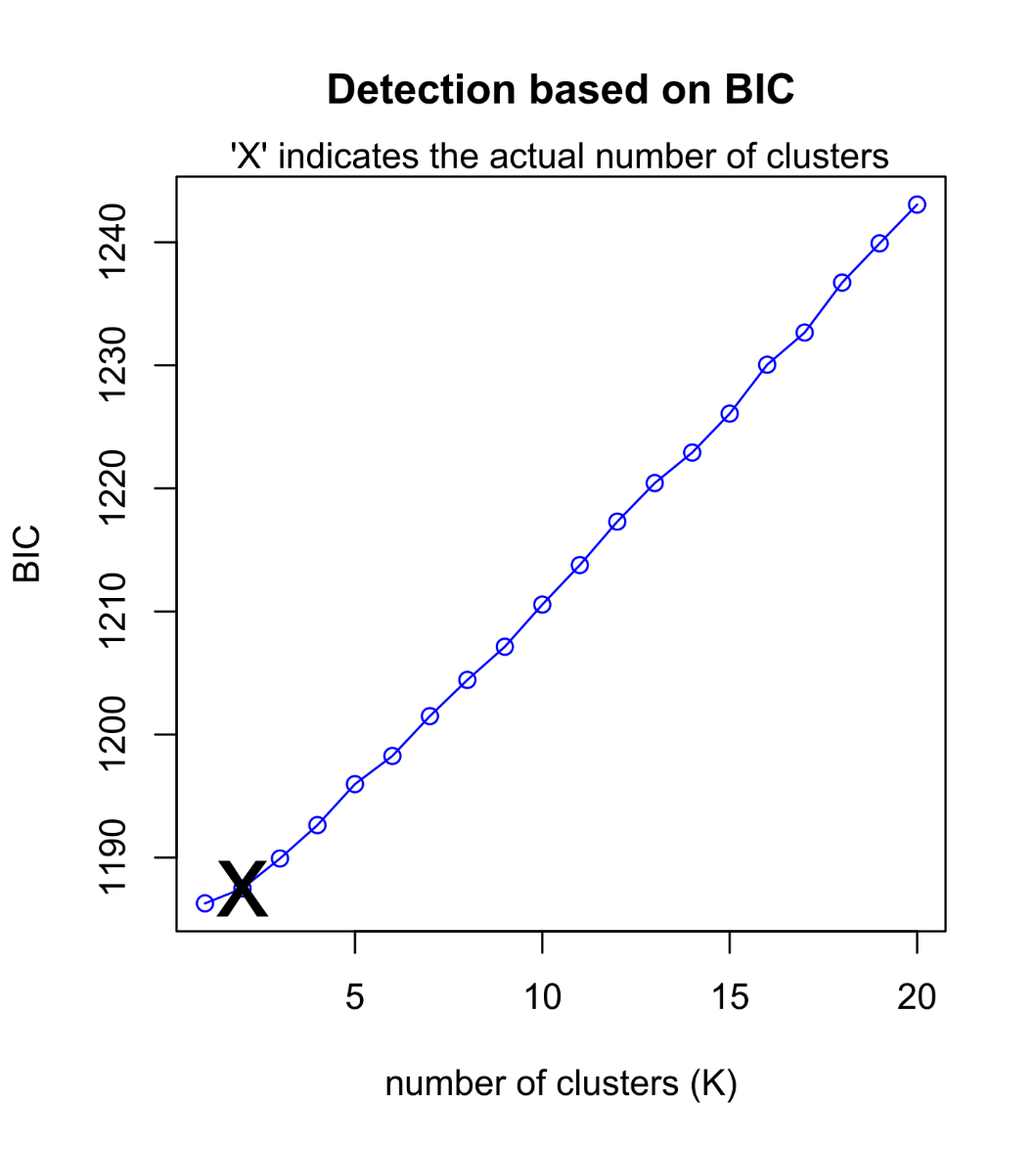
****Supplementary data 3**: Bayesian Information Criterion calculated by adegenet running an unsupervised clustering approach using Discriminant Analysis of Principal Components in adegenet (DAPC, Jombart et al., 2010) by running successive K-means, without assuming a panmictic population. The number of populations explaining the observed genetic variation best is K =2.

**Supplementary data 4**: Summary statistics of negative binomial GLMM for the abundance of *Deuteragenia subintermedia* and the selected environmental variables with the Regions (Hochschwarzwald, Südschwarzwald, Mittleres Rheintal, Hochrhein, Baar/Hegau) as random factor.

| **Environmental variable** | **Estimate** | **Std. Error** | **z-value** | **p-value** |
| --- | --- | --- | --- | --- |
|  |  |  |  |  |
| Average DBH | 0.052 | 0.070 | 0.750 | 0.453 |
| Average elevation | -0.009 | 0.064 | -1.42 | 0.156 |
| Basal area | 0.019 | 0.071 | 0.027 | 0.079 |
| **Canopy closure** | **-0.131** | **0.062** | **-2.140** | **0.032** |
| Effective number of layers | 0.002 | 0.064 | 0.050 | 0.964 |
| Forest cover 10 km^2^ | -0.045 | 0.057 | -0.800 | 0.426 |
| Lying deadwood (log) | 0.030 | 0.060 | 0.500 | 0.616 |
| Percentage coniferous | 0.110 | 0.065 | 1.680 | 0.093 |
| Standing deadwood (sqrt) | 0.066 | 0.055 | 1.200 | 0.232 |

**Supplementary data 5:** Summary statistics of averaged model based on negative binomial GLM, from model selection using the Akaike Information Criterion (AIC) retaining models with a delta AIC < 2, for the abundance of *Deuteragenia subintermedia* and the selected environmental variables.

**Model averaged coefficients:**

| **Environmental variable** | **Estimate** | **Std. Error** | **z-value** | **p-value** |
| --- | --- | --- | --- | --- |
|  |  |  |  |  |
| Average DBH | 0.04 | 0.020 | 0.183 | 0.828 |
| Average elevation | -0.022 | 0.048 | 0.459 | 0.646 |
| Basal area | 0.002 | 0.016 | 0.105 | 0.917 |
| **Canopy closure** | **-0.123** | **0.057** | **2.138** | **0.033** |
| Effective number of layers | 0.004 | 0.020 | 0.190 | 0.850 |
| Forest cover 10 km^2^ | -0.008 | 0.028 | 0.291 | 0.771 |
| Lying deadwood (log) |  |  |  |  |
| Percentage coniferous | 0.034 | 0.057 | 0.605 | 0.545 |
| Standing deadwood (sqrt) | 0.005 | 0.024 | 0.216 | 0.828 |

**
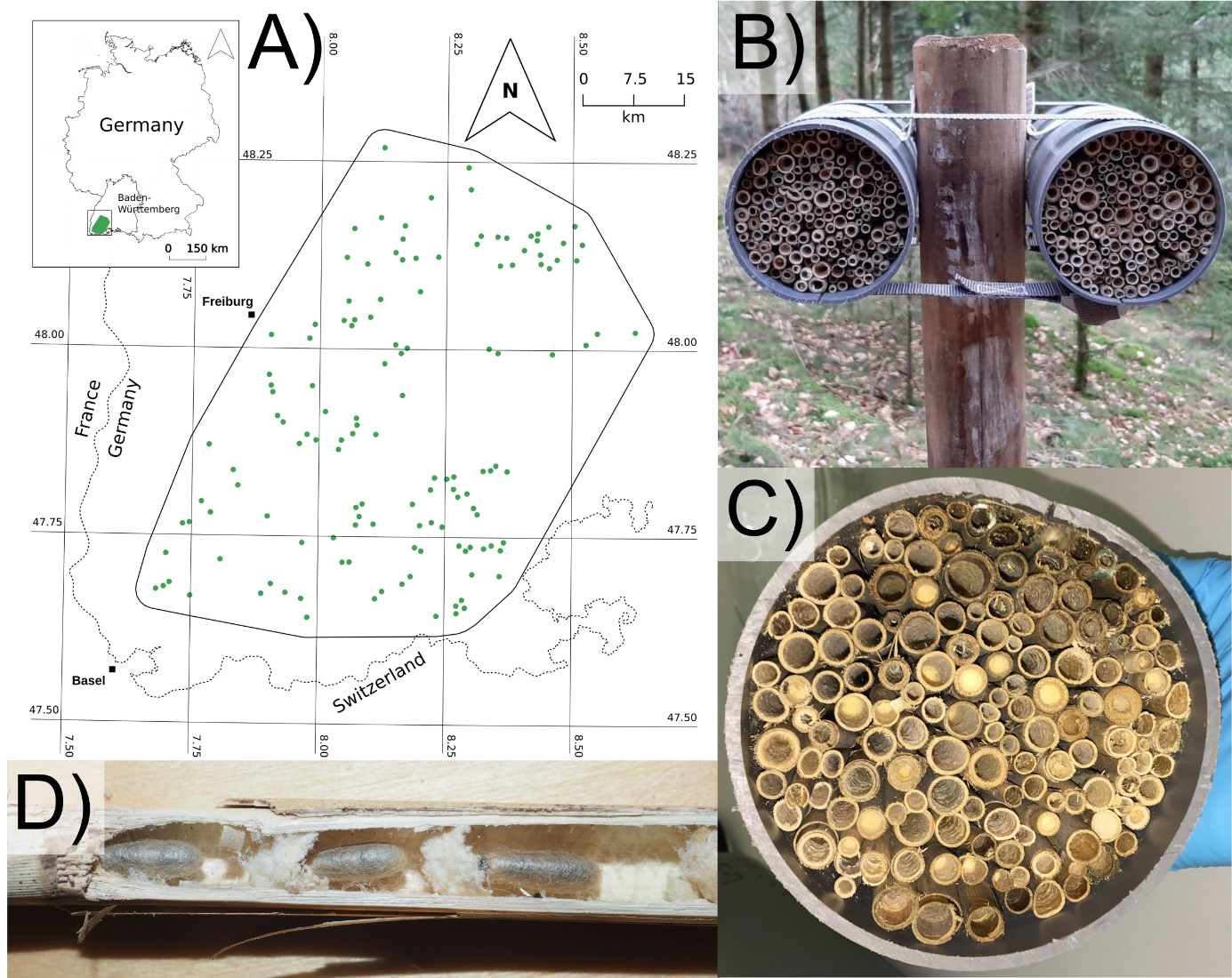
****Supplementary data 6:** Sampling overview **A)** Location map showing the research area of the ConFoBi project with the 135 research plots marked as green dots, for further details see Storch et al. 2020 https://doi.org/10.1002/ece3.6003 **B)** Photograph of trap nest used for collection, directly after installation **C)** Photograph of trap nest after collection at the end of the season, with reeds plugged by different species of cavity-nesting Hymenoptera **D)** Photograph of a reed cut open along its length, showing nesting cells of *Deuteragenia subintermedia*
